# On the Use of Mercury in Diseases of the Liver

**Published:** 1802-09-01

**Authors:** J. B. Davis

**Affiliations:** Tower, London


### *On the Use of Mercury in Diseases of the Liver; communicated by Mr.* J. B. Davis, *of the Tower, London.*

MERCURY deſervedly holds a very high rank in the removal of chronic Obſtructions of the Viſcera, and I believe that I am not ſingular when I aſſert it is our ſheet anchor in the cure of the Schirrous Liver.

Previous, however, to ſpeaking of its beneficial influence on the various affections of that organ, I have taken the liberty to trouble you with a few precurſory obſervations relative to the diſeaſes of the hepatic ſyſtem. I think it too the more neceſſary to do this, as we are apt, from the common occurrence of bilious complaints, to diſregard many pains of the liver, upon a ſuppoſition of their being merely ſympathetic. We muſt all acknowledge, that no part is more eſſential to the well being of the animal economy than this; of courſe, then, none claims our attention with ſtricter propriety. Pain is often the only characteriſtic ſymptom of diſeaſe, as in cephalalgia, odontalgia, gaſtrodynia, arthritis, ſplenalgia, hepatalgia, &c. But what advantage, I would beg leave to aſk, will accrue to practice from ſuch information, unleſs the preciſe ſituation and probable cauſe of it be both thoroughly inveſtigated. Hippocrates ſays, "That whatever eſcapes the reach of our external ſight, ſhould be ſearched for, and overtaken by the eyes of the mind." Of what uſe is it to know that there is pain in the hypogaſtric region, if we do not at the ſame time learn whether it proceeds ceeds from inflammation, obſtruction of the biliary ducts, or ſchirroſity. The neceſſity of ſuch a diſcrimination is the more ſtriking, when we reflect that a different mode of treatment is required in each. The ſize of the liver, and the collective area of veſſels containing blood for the ſeparation of bile, ſhew how large a quantity is carried there for that purpoſe; and if we only recollect the conſtant ſupply of bile required for chylification, and which indeed, in a ſtate of health, is continually going into the duodenum, it is a matter of perfect aſtoniſhment that the pori biliarii, owing to the tenacity of the ſecerned fluid, do not become more frequently obſtructed. The bile muſt flow uninterruptedly into the chylopoietic viſcera, as a conſtriction of the ductus choledochus has often painfully evinced.

If the healthy functions of the liver be not *suddenly* ſuſpended, and a part of its ſecretion flows through the proper channel into the duodenum, and another portion, comparatively ſmall, out at any external opening, ſtill the moſt vigorous animal would gradually decline, and in the event probably die. So true it is what Lord Bacon ſays, "That the bile is the incentive and ſtimulus of many functions of the body." Wounds in the gall bladder likewiſe convince us, how large a quantity of this fluid is ſecreted; and they alſo prove that the cyſtic bile, though not ſo conſtantly paſſing into the duodenum, cannot be loſt without equal danger. Without this uſeful ingredient the inteſtines no longer perform, with energy, their periſtaltic motion, the heterogenous ſubſtances contained in the aliment do not unite, nor is the chyle duly mixed for the reception of the lacteals, or the nutrition of the animal.

The qualities of the bile ſhould be thoſe of a natural ſoap, intimately combining with the oily and watery particles of the chyle in its paſſage through the duodenum; but when the blood has been heated and rendered acrimonious by hard drinking, violent exerciſe, or warmth of climate, the properties of it are ſo much changed, and rendered ſo very ſtimulating, that inſtead of anſwering ſuch a ſalutary end, it will infallibly create pain and irritation.

In the event of ſpaſm of the ductus choledochus, Nature relieves herſelf by an abſorption of the finer particles; but this effort, unleſs the uſual channel is ſoon reſtored, will be inadequate to the ſuſpenſion of head-ach, vomiting, pain, and other equally diſtreſſing ſymptoms. A warm climate neceſſarily heightens the alkaleſcent particles of the bile; but they are doubtleſs made much more ſo by a too liberal uſe of ardent ſpirits, and a frequent ſtoppage of ordinary perſpiration from whatever cauſe it may proceed.

Nothing

Nothing is ſo difficult as to perſuade hard drinkers to forego their favourite purſuit; and if they ſhould even have courage to improve by the hint, it is more than probable, that the cuſtom would have already laid the baſis of that diſeaſe, which will ever after require the greateſt care on their part, and the ſkill of the moſt judicious phyſician, to keep within bounds.*

——— Eripe turpi

Colla jugo, liber liber ſum, dic age. HOR. SAT. 7.

——— Video meliora proboque

Deteriora ſequor. OVID. MET.

Some people are ſo liable to a ſuffuſion of bile, that cold, irregularity of any kind, or trifling indiſpoſition, checking the healthy actions of the conſtitution, would immediately bring on an attack preceded by ſevere pains in the ſide. Others, who are ſubject to gall-ſtones, are, from the ſame ſuſceptibility, ſcarcely ever free from local pain and irritation. The latter acting upon the hepatic ſyſtem, is, agreeably to the doctrine of Doctor Gottlieb Richter, the cauſe of icterus, and may ariſe from concretions, ſpaſm, inflammation, or whatever impedes the flow of bile through the pori biliarii. A ſuffuſion of this kind is for the moſt part of temporary duration, and the ſurcharge which the blood ſuſtains ſoon carried off by the ſkin or bowels. When an obſtruction is allowed to remain, the finer particles gradually find their way into the circulation, and thus a niſus is left for the formation of a ſtone. During this time, I conceive the ſymptoms of jaundice may recur very often, and with equal ſeverity, as if a ſmall gall-ſtone was paſſing through the duct.

Spaſm of the ductus choledochus is frequently very acute, and aſcertained by being deeply ſeated between the ribs and and dorſal vertebræ. It was the practice of the celebrated Hoffman to bleed at the commencement of an attack, purge, give antimonials, ſoluble and cream of tartar, diluents, &c. and it appears indeed, from experience, to be the moſt judicious treatment which could poſſibly be deviſed.

The great deſideratum, on the acceſs of ſevere ſymptoms, is the removal of pain and irritation, events not very likely to happen without firſt making liberal evacuations. All irritants, therefore,

* Medicine can be of but little ſervice, when patients obſtinately perſiſt in ſuch an injurious practice. The Grecians were very ſtrict with reſpect to the conduct of ſick people. " A capital puniſhment uſed to be inflicted by the Locrians on the patient for diſobedience to the advice and orders of his phyſician." So great was the reverence ſhown to the healing art in all Greece, and at Athens in particular.

fore muft, for obvious reafons, be precluded till tenfion is removed, and then they may probably be prefcribed with great advantage. A calomel purge is ufeful in the beginning, and may be adminiftered with perfect fafety. This, however, is not the affection of the liver wherein mercury is of fuch decided fervice, though I have known it do a great deal of good towards the end of the paroxyfm. It is defervedly held in high eftimation as an alterative, and with this view may be prefcribed in fmall dofes, to be taken every night for a fortnight or three weeks, then difcontinued, and after a proper interval refumed, taking care to carry off its effects with caftor oil or Epfom falts, once in five or fix days. I think a grain of calomel, a grain of ipeçacuanha, and two grains of foap, is an excellent compofition.

By this plan, and a fuitable mode of living, a recurrence of the complaint is not unfrequently fufpended for many months. At the heighth of fpafm, laudanum ftands unrivalled, and ought certainly to be exhibited till the violence of pain abates. Now and then the ftomach is too irritable to retain it, in either a folid or a liquid form, and then I have generally had recourfe to the extract of poppies, which has oftener than once fucceeded in allaying the uneafinefs of the ftomach, and overcoming fpafm. This extract is not generally ufed in practice; a circumftance which I much regret, as it would be found a ufeful fubftitute in thofe cafes wherein opium was rejected or difagreed with the patient's conftitution.

> " Tho' mixt with wheat I grow,
> Indulgent Ceres knew my worth,
> And to adorn the teaming earth
> She bad the poppy blow.
> Seize, happy mortal, feize the good;
> My hand fupplies thy fleep and food,
> And makes thee truly bleft;
> With plenteous meats enjoy the day,
> In flumbers pafs the night away,
> And leave to Fate the reft."

There are other bilious perfons who cannot take the lighteft food without fuffering internal heat, flatulence, ficknefs, and occafionally pains in the head and fide. Their fkin commonly has a yellow tinge, and the tunica conjunctiva partakes of the fame hue; but I have remarked that thefe people are feldom fo ill as to have a regular attack of jaundice, or be ferioufly troubled with gall ftones.* The circulation is very languid in them, and the bile remarkably thick. The fluctuation of fpirits which thefe patients are fo liable to, treating their com-

* ——— " Facies non omnibus una
Nec diverfa tamen."

plaints with indifference at one time and ferioufnefs at another, gives them the character of Hypochondriacs; yet in no other refpect do they particularly refemble thofe unfortunate Beings. I have no doubt of their being fome fault in the bile, though I cannot avoid thinking that "ægrotant animo magis quam corpore."

Exceffes are very injurious in *a healthy conftitution*, and much more fo in habits like thefe, already reduced by irritation and mental inquietude. Private entertainments, fumptuous feafts, long walks, then fudden abftemioufnefs and perfect reft, are all equally injurious, and will, by gradually overcoming the natural elafticity of the folids, fooner or later, render the fluids unfit for the various purpofes of the animal œconomy.

> Cum labor extuderit faftidia, ficcus, inanis,

> Sperne cibum vilem; nifi Hymettia mella Falerno

> Ne biberis diluta. Hor. lib. 2.

I coincide with Dr. Cullen, in its being an object of the firft confideration, to amufe the mind at the time you are ftriking at the root of the real malady. The patient may have unneceffary fears, and then treat his true complaint with altogether as much levity; but be that as it may, the Practitioner will obtain great advantage by allaying mental irritation. Idle perfons, who fare luxurioufly, and are irregular in their general mode of living, are thofe to whom I allude, and not fuch as are engaged in fedentary employments, or have had their fpirits broken by frequent troubles and difappointments.

In all thefe cafes, in my poor opinion, regular exercife ought to be enjoined with every perfuafive argument which the medical attendant can feafonably apply.* I have witneffed the recovery of feveral patients by fending them fome diftance, three or four times a week, to drink the water of any chalybeate fpring. The ftomach is thereby braced, the mind amufed, and the circulation reftored. The medicines which fucceed beft in thefe cafes are warm cathartics, fmall dofes of calomel, ipecacuanha, and rhubarb. The habit being thus previoufly corrected, colomba, gentian, chamomile flowers, quaffia, aloetic wines, and other ftomachics, ftrengthen the tone of the vifcera, and promote healthy fecretions. If fpafm fhould attack the ductus choledochus, or pain take dominion of the liver, neither of which are very uncommon in a confined ftate of the bowels, I have almoft always procured temporary relief from Hoffman's anodyne

* The ancients attributed the greateft utility to exercife properly regulated, and fo indeed do the moderns. The great Sydenham declares, "that mercury for Lues Venerea, nor bark for the Intermittents, are not more certain fpecifics than riding on horfeback for a confumption." What benefit then are not bilious people likely to derive from moderate bodily exertion!

dyne liquor, and a fmall quantity of luadanum; or, if thefe failed, camphor, julep, and fpt. ammon co. After fomenting the fide with a decoction of poppy heads and chamomile flowers, a warm plafter put upon the right hypochondrium often procures eafe. Sometimes an embrocation of fpt. camph. tinct. canth. and aq. ammon. does more good.

A diet confifting of plain wholefome food, and after dinner two or three glaffes of generous wine, provided the patient has long been in the habit of taking it, will be more conducive to health than a catalogue of the moft valuable compofitions. If you do not choofe to place your whole dependence upon it, you fhould at leaft take care that it is fuch as will affift the operation of medicine, or in no fhape whatever counteract its effects. Galen afferts, that "That medicine has no efficacious remedy which can bring any permanent affiftance if the mode of living fhould refift it, or fhould not act in conformity, and become an ufeful auxiliary. By diætetic remedies, he fays, that he has overcome the moft obftinate chronic difeafes, which have been treated in vain by medicines elegantly compounded, and judicioufly adminiftered. This correfponds with the old adage,

"Cur Moriatur homo, cui Salvia crefcit in horto."

Piles are not unfrequently the confequence of an obftructed egrefs of the bile, and fhould therefore be repelled with the greateft caution.

As the natural channel is reftored by the means before mentioned, the hæmorrhoids ceafe or recur but feldom. They are entirely fymptomatic of fome accumulation in the liver,* and are preceded by a fevere pain in the fide, which, for the moft part, abates as hæmorrhagy comes on.

When once the functions of the liver have been perverted, and morbid action gained a gradual afcendency, the hepatic fyftem, like other parts of the human body, though completely reftored to its original ftate, is ever after liable to relapfe; a circumftance which the patient as well as practitioner fhould be well aware of. Inattention to this point have led many to fay, in a fhort time, with Juvenal,

"Plus aloes quam Mellis habet."

Gall ftones generally excite a vaft deal of pain in their way to the duodenum, and that, in proportion to the irritability of the ductus choledochus. I have known concretions, of a peculiar kind, voided, which produed very little uneafinefs;

* I do not mean by this inference to affert that fuch an accumulation is the general caufe of piles.

but whenever the calculous matter is rough and hard, it cannot fail of exciting confiderable pain. The head is often feized with fhooting pains, and a fenfation of fomething girting it, and the diaphragm has momentarily ceafed to act from the irritation of a fmall quantity of fine gravel or fand.

I am acquainted with a lady who is attacked almoft every month with a violent head-ach, pain in the fide, and vomiting, which continue two or three days, fometimes longer, and then gradually fubfide as the calculous matter is rejected from the ftomach, or carried off by the bowels. A dark fubftance, of an irregular figure, is now and then brought up in the act of vomiting. Until this is removed fhe finds no eafe, and laudanum, the fine qua non for the mitigation of fpafm, almoft distracts her. Mercurials and the whole lift of antibilious medicines have been tried, with the Bath, Cheltenham, and Tunbridge waters; but all, I am forry to fay, to little purpofe. The moft relief has been procured from calomel and foap combined, ftimulating liniments to the fide, and brifk purges. The bowels are always torpid, and require ftrong aperients. This lady was inclined to be bilious at a very early period of life; and ever fince her return from the Weft Indies, where fhe refided two or three years, fhe has never been a month free from this vexatious difeafe. The climate of England is generally ferviceable to people in fimilar fituations, but, unfortunately, it has no beneficial influence upon her.

Of all the difeafes of the liver, fchirrus is the moft to be dreaded, the functions of that organ becoming permanently impeded, and the fyftem cachectic. It is flow in progrefs, and always accompanied by an obtufe pain under the ribs. The face, if before florid, acquires a dark yellow hue, the appetite diminifhes, breathing is performed with difficulty, the bowels become torpid, and the legs fwell. "Ubi hepar fchirrofum eft, fæpe fit fanguinis curfus ex naribus, gingivis, et umbilico; vomitus etiam fanguinis eft, et alvus reddit cruenta*." Other affections of the liver, excepting thofe of a fcrophulous defcription, are generally temporary, and yield as the natural channels are reftored. The conftitution alfo improves in the intervals; but in the fchirrous liver there is feldom any amendment. It gets worfe by degrees, and is at laft perceptible to the touch†. "Hi paucis menfibus moriuntur, illi annos aliquot fuperfunt; plerique paulo ante mortem fiunt hydropici."

Mercury is the only remedy which can cope with this difeafe;

* Heberden de Hiftoria et Curatione Morborum.

† Ibidem.

eafe; and to every other medicine, in fhort, it feems to bid defiance. Some practitioners depend upon internal dofes, but for my own part, I would rather at once have recourfe to friction. Leeches may be firft proper; and if there was much pain, I fhould certainly wait, till, by topical bleedings and general evacuations, I had abated the violent action, and thereby rendered the exhibition of mercury perfectly fafe. Ten or twelve leeches might be applied two or three times before the ointment was ufed. If the patient's ftrength would admit of it, neither the cupping-glafs nor leeches fhould be fpared.

The fkin being generally hot, and the pulfe accelerated, nitre, fal polychreft, antimonials, and gentle aperients may be prefcribed with the greateft advantage. If the cafe be complicated with a lodgment of water in any of the cavities, elaterium, cream of tartar, and mercurial purges muft not be omitted, though in fuch an advanced ftage of the difeafe there is but little chance of reftoring health! No harm can refult from an early ufe of mercury; for fhould it not prove to be the identical complaint under confideration, the fyftem would in all probability derive benefit from the action of fo difcutient a medicine; but if, on the contrary, the moft irremediable ills will occur if the adminiftration of it be long protracted.

A gentleman who was in the Weft Indies caught the yellow fever, and has fince had a fchirrous liver. He took a variety of medicines, and amongft others calomel and cicuta, which always gave him relief to a certain degree. In hopes of regaining his ufual health from the air of England, and a temperate mode of living, he waited that event for feveral months with great patience and refignation; but being difappointed in his wifhes, at the end of fo long a period, he refolved, without farther delay, to enter upon a regular courfe of medicine. He was under the action of mercury three or four months, during which time an evident amendment took place. Sometimes a drachm of the ung. hydrarg. fort. with camphor was rubbed into the fide every night, then half a drachm, and fo on, taking care neither to overload nor relax the conftitution too much by fo free an exhibition of it. I fhould have mentioned that leeches were applied two or three times, and ten grains of nitre given every four hours. Soluble tartar and caftor oil were ufed as occafional aperients.

This gentleman, to all appearance, got perfectly well in about four months, and by living abftemioufly in a pleafant part of the country, and taking regular exercife, has, to the prefent day, efcaped a return of the complaint. As his chief ftudy is the recovery of health, and his circumftances very eafy, I have no doubt of this plan effecting a complete reftoration.

"Beatus

"Beatus ille, qui procul negotiis,

Ut prifca gens mortalium,

Paterna rura bobus exercet fuis

Solutus omni fœnore."— HOR.

Tubercles are fuppofed to be peculiar to hard drinkers, and although flow in growth, acquire confiderable magnitude.* In fcrophulous fubjects alfo, indurations of a remarkable fize have been frequently found. The only criterion by which this affection may be diftinguifhed, is the prevalence of ftrumous action at the fame time in fome other part of the conftitution, or the veftiges of its former influence. Pain in the fide and cachexy are common accompanyments. Mercury affords *trifling relief* in the latter difeafe, but feldom, I believe, does more: It is, however, the only remedy which holds out a profpect of fuccefs; and although the complaint may be incurable, you may have the good fortune, by an occafional ufe of this valuable medicine, to render it ftationary, and thereby enjoy the fatisfaction of having prolonged life.†

I avoid faying any thing upon hepatitis, as mercury, from its ftimulating property, can neither be a fafe nor advifeable medicine in the cure of it.

*August*, 9, 1802.

* I have been prefent at the opening of bodies wherein I have feen fix or feven tubercles in the fame liver. In a middle aged man, three tubercles of the fize of a fmall apple were taken out, which, when diffected, had a firm and ftriated texture. In another fubject, a remarkably large one was removed, of a much fofter confiftence; and upon the anterior edge of the fame liver I difcovered three or four more fmall tumours.

† A morbid thickening and hardnefs of the liver is always relieved by mercury.

